# Real-Time Assessment of Causal Attribution Shift and Stay Between Two Successive Tests of Movement Aids

**DOI:** 10.1007/s12124-020-09592-7

**Published:** 2021-01-09

**Authors:** Takumi Ohashi, Makiko Watanabe, Yuma Takenaka, Miki Saijo

**Affiliations:** 1 Tokyo Institute of Technology, 2-12-1 O-okayama, Meguro-ku, Tokyo, 152-8550 Japan; 2 National Graduate Institute for Policy Studies, 7-22-1 Roppongi, Minato-ku, Tokyo, 106-8677 Japan; 3 Amazon Web Services Japan K.K., 3-1-1 Kamiosaki, Shinagawa-ku, Tokyo, 141-0021 Japan

**Keywords:** Attribution theory, User experience, Technology acceptance model, Utterance analysis, Frail elderly

## Abstract

The development of welfare assistive devices for frail elderly people has attracted significant attention for its effort to improve the quality of life and reduce the burden on caregivers. However, it is challenging to conduct multiple user tests because of the significant burden on the elderly; thus, we need efficient ways to extract insight through different approaches. In this study, we aim to elucidate real-time transitions in users’ emotions and achievement motivation while using such a device. We synthesize an utterance analysis method based on attribution theory, in which all users’ utterances are attributed to four categories (ability, effort, task difficulty, and luck) that follow the developed coding rules. Knowing the transitions in causal attribution allows us to extract salient experiences for users, especially by extracting shifts from them and analyzing why the shift occurred and what exactly was happening before and after the shift. If only salient user experiences can be referenced from the aggregate data, useful information can be extracted in a short time to improve system characteristics and the environment. We discussed the validity and reliability of the proposed method by conducting a user test of an electric-assisted four-wheeled cycle for frail elderly people in Kakegawa city in Shizuoka, Japan. We also succeeded in marking the points that need attention, which is about 33% of the total amount of utterance data (1626 utterances), and thus confirmed the potential of the proposed method. Future research should examine how the developed methodology can help designers improve assistive device development, as well as how it can contribute to other fields such as education and social assistance.

## Introduction

To reduce social security costs for the elderly and simultaneously ensure their well-being, welfare devices have attracted considerable attention in recent years from the following perspectives: (1) supporting caregivers and (2) assisting/controlling the deteriorating cognitive and physical functions of the frail elderly (people who need care) (Schulz et al. 2015; WHO 2015). In this paper, within the group of medical and welfare equipment, we define “welfare device” as a device that assists physical functions and encourages social participation. Regarding the second perspective, it is known that physical activity (such as moderate exercise) leads to the reduction of several types of risks (Laurin et al. 2001). Therefore, a device that assists the frail elderly in their activities is expected to keep them active. However, encouraging the frail elderly to undertake such an activity is not an easy task.

**Fig. 1:**
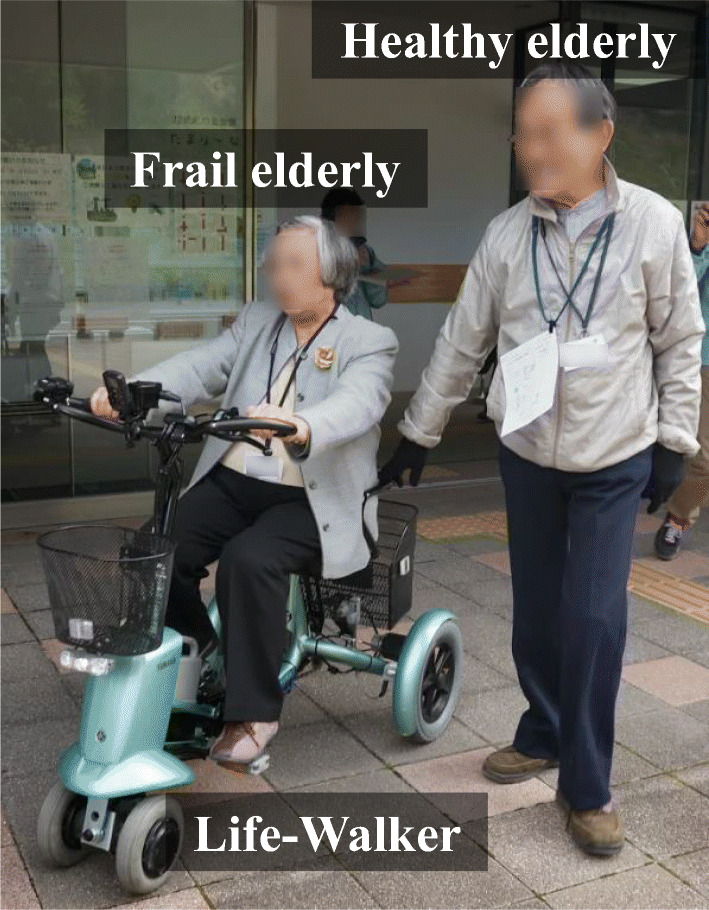
A user test of the Life Walker in Kakegawa City, Shizuoka Prefecture; a frail elderly person is assisted by a healthy elderly person

In the authors’ research group, we have accumulated research on ways to encourage the frail elderly to engage in physical activities while enhancing their quality of life (QoL) according to an “enjoyable outing” concept, using an electric-assisted four-wheeled cycle called the Life Walker (by Yamaha Motor Engineering Co., Ltd., as seen in Fig. 1). This is an example of a welfare device that was developed to allow users with disabilities to move as fast as people who can walk (Ohashi et al. 2017; Saijo et al. 2014; Watanabe et al. 2020). Figure 1 shows a user test of a Life Walker in Kakegawa City, Shizuoka Prefecture (more details in Section 4). The frail elderly tend to lose their connection to society even though, in some cases, they successfully engage in communication with healthy people. This type of connection with society plays an important role in maintaining psychological well-being (Kawachi and Berkman 2001). For this reason, we expect welfare devices to not only physically support activities, but also serve as a tool for communication (Ohashi et al. 2017). However, a welfare device does not make any sense if it is not used. Therefore, it is necessary that we design devices and environments (including caregivers’ behavior, etc.) that can be used in the frail elderly’s daily life.

Although frail elderly people are not the only ones prevented from participating in society because of a loss in physical functions, everyone inevitably loses body functions with age, and therefore the mass market segment for welfare devices undoubtedly consists of the frail elderly and their reserve group. A significant challenge in developing welfare devices is the extreme difficulty in carrying out repeated user tests because of the burden caused and the fact that the reliability of users’ introspection is estimated to be lower than that of other user groups (due to age-related loss of cognitive function). It is necessary to not only extract user needs in an efficient manner, making use of limited opportunities for user tests, but also assess the reliability of extracted needs using a method that does not rely solely on introspection. Such an approach would help improve assistive devices, and provide the frail elderly with a device that can be used continuously.

In conventional user tests, methods such as paper questionnaires, retrospective interviews, and observations by specialists have been used to extract potential user needs. However, these methods require a certain number of prior assumptions regarding user needs, and it is difficult to extract findings outside the scope of such assumptions. In addition, a method called “think aloud” exists for extracting “momentary” thoughts from the user during user tests, but this imposes a significant burden on the frail elderly. Therefore, methods of extracting introspection from users during or after user tests are not suitable in this case (as the adaptability is low).

Thus, in this research, we try to elucidate users’ experience of testing the product by extracting the transitions (i.e., shifts and stays) in users’ emotions and achievement motivation in real time, without relying on introspection. To do so, we focus on the utterance data collected during tests. Using attribution theory, we developed a method in which all utterances are assigned to four categories, and the transitions of these attributions are used to estimate changes in achievement motivation. To verify the proposed method, we conducted user tests with a welfare device (an electric-assisted four-wheeled cycle: Life-Walker) used by pairs made up of a frail elderly person (driver) and a healthy elderly person (caregivers) in Kakegawa City (Shizuoka Prefecture).

## Theoretical Background

### How People Accept Technology: Existing Research on Models of Technology Acceptance

Research on people’s technology acceptance has been conducted in various contexts, including in the context of welfare devices for the elderly.

The Technology Acceptance Model (TAM), which was proposed by Davis (1986, 1989) to explain individuals’ information system acceptance, has been widely utilized in the realm of information systems, as well as many other fields; TAM is one of the behavior intention models developed to explain user system use. In addition, many literature reviews on papers that model technology acceptance/dissemination have been conducted, indicating the existence of various other models (e.g., Alkhwaldi and Kamala 2017; Lai 2017). However, Abrilahij and Boll (2019) pointed out that existing models which combine various predictors have less explanatory power for “intention to use” and “actual use” because the models do not consider some of the important predictors of users’ mental representations. What is more, they state, from an action-theoretical perspective (e.g., Brandtstädter 2006), that it is necessary to use both interrelated variables of belief and desire to explain the action (e.g., use of an assistive device).

Although models of technology acceptance have been developed as described above, the predictors are very broad concepts. For example, when we think of the predictor “perceived difficulty to use” in the models for assistive device use by the elderly, what specific part of the assistive device is difficult for them to use? When we think of “privacy concern,” what specific part of the assistive device raises such privacy concerns? Lee et al. (2003) noted that, while the simplicity of TAM may attract researchers, practitioners will not be impressed and will be treated badly (i.e., they will feel useless). The reason is that the TAM does not provide specific guidelines for designing good systems or choosing from competing systems. In other words, many models give us clues on why elderly people would or would not use a device, but in order to effectively improve elderly people’s daily use of devices, we need a certain method that can break down these clues into more detailed points. The present study focuses on causal attribution, which has been neglected in technology acceptance models. The causal attribution theory, discussed later in this paper, focuses on what causes one can attribute to success and failure in the achievement of the task. By applying this theory, we can understand exactly what elderly people attribute to their successes and failures during device use. If we know that users attribute the cause of failure (or success) to a particular aspect of the device, we can focus on improving (or reinforcing) that particular aspect.

### Technology Acceptance from the Human-Computer Interaction (HCI) Perspective

The research on technology acceptance, which emphasizes the utilitarian aspect, points to the importance of combining a user experience (UX) model in the context of human-computer interaction (HCI) research, such as the introduction of experiential factors (e.g., hedonic factors such as perceived enjoyment), with factors that change over time (Hornbæk and Hertzum 2017). According to Hassenzahl and Tractinsky (2006), UX is a result of a user’s internal state (e.g., expectation, needs, feelings, emotions), the designed system features (e.g., complexity, objective, usability, functionality), and the context and environment that create a mutual effect (e.g., organizational/social conditions). Although few studies simultaneously examine the two models, because of the difference in the development history of each (Hornbæk and Hertzum 2017), it is similar to the definition of UX above in that various TAM studies examine users’ mental representations of themselves, the assistive technology, or the situational context (Abrilahij and Boll 2019). Thus, extracting insight from two different research fields, where each focuses on the utilitarian aspect and the experiential aspect respectively, may lead to a more solid understanding of user-technology interactions.

According to Hornbæk and Hertzum (2017), who have reviewed papers that tackle UX models and TAM simultaneously, factors such as perceived enjoyment and usefulness seem to determine and precede the use of the system/technology in various models, but at the same time these factors are affected by use of the system. This suggests that the intention to use a system is continuously formed and changed based on how useful the current system is to the users, and how much pleasure the users feel in the given moment. Although Agarwal and Karahanna (2000) retrospectively measured the state of cognitive absorption in their research, they also mentioned that “an ideal examination of this state would be during the activity that is its cause (i.e., the human-computer interaction), or immediately following the activity.” This suggests a need for a methodology that estimates a user’s internal state in the moment, in order to effectively consider changes over time.

### Conventional User Test Problems for the Frail Elderly

In contexts that involve trial products or prototypes, what type of user tests should be conducted to efficiently extract transitions in a user’s internal state? In the User Experience White Paper (2011), UX is divided into four phases from a time-based perspective: anticipated UX, momentary UX, episodic UX, and cumulative UX.

To grasp detailed transitions in users’ internal state, such as emotion related to a device, it is necessary to focus not only on memory but also on UX during use (momentary UX). “Think-aloud” is one of the major methods for extracting momentary UX. In this method, users who tackle an issue related to the test are asked to say everything that arises in their minds—feelings, thoughts and understanding—and the outcome is recorded. This method is used for the purpose of monitoring the thinking process and is supposedly adequate for extracting transitions in users’ internal state. In contrast, in some cases, the user ignores the instructions and performs actions in silence. Moreover, it is necessary for the user to master the method in question; as such, this method is considered to represent a significant burden for the frail elderly.

Transitions in internal states can be perceived only by the specific target themselves (in some cases, even the person in question is unaware of it). From this standpoint, it is possible that utterance data contains real-time information about users’ experience (momentary UX) while tackling the issue. Therefore, it is reasonable to focus on utterance data, which is an expression of a user’s own introspection. It is necessary to develop a simple user testing method that does not place a burden on the user, and does not require the user to master the task. In this paper, we try to develop a simple methodology, while also validating the conventional method (i.e., questionnaire survey, observation method).

### Application of Causal Attribution Theory: Analytical Advantage in a Device Development Context

The internal state of the elderly changes over time because of various events while using the device, and this state, especially in terms of emotions, is very complex (Cowen and Keltner 2017). There are many studies that attempt to extract emotions from facial expressions (e.g., Li and Deng 2018; Mollahosseini et al. 2019) and brain activity (e.g., Gerber et al. 2008; Jirayucharoensak et al. 2014; Mohammadi et al. 2017). However, even if changes in emotional fluctuation can be detected over time by continuous measurement of facial expressions and/or brain activity, it is difficult to estimate what the fluctuation is caused by, particularly in terms of whether it is due to internal or external factors (e.g., environment, devices).

In this context, the present study focuses on the subconscious process, namely, the causal attribution that affects people’s emotions. After an event, people unconsciously explain (attribute) the cause of the outcome of an event, and this attribution acts on emotions and ultimately raises motivation for the future task (Cook and Artino 2016), namely achievement motivation. In Weiner’s (1985, 2014) attribution theory, the perception of three main cause-and-effect relations are mentioned: locus of causality (causes perceived as residing either within or outside the person), stability (causes perceived as stable or unstable), and controllability (causes that the agent may or may not change). These three dimensions of cause-and-effect influence several common emotional experiences (anger, gratitude, remorse, despair, pride, shame, etc.), and depending on the attribution of such facts or results, the expectation or emotion is influenced, which consequently defines the directions of actions. Here, it should be noted that Weiner et al. (1971) state that controllability can be distinguished from other dimensions based on experience, but they may not be orthogonal. Therefore, we have excluded controllability from this research. As shown in Table 1, we only adopted two dimensions (stability and locus of causality) and four perceived determinants of achievement behavior (effort, ability, task difficulty, and luck).

**Table 1:** Systematic classification of the perceived determinants for actions related to the accomplishment of a task (Weiner et al. 1971)

Locus of causality	Stability	
	Stable	Unstable
Internal	Ability	Effort
External	Task difficulty	Luck

Conventionally, attribution theory has been used to explain mid- to long-term individual tendencies. We, on the other hand, attempt to apply attribution theory to real-time assessment, considering that every moment ultimately shapes individual tendencies. In this study, we classify utterances obtained from user tests into the causal attribution quadrant. For example, if users believe that what they are doing is not working, we classify from their utterances whether they are trying to attribute the cause to something internal and stable (i.e., ability) or something external and unstable (i.e., luck). In this way, all utterances are classified as a specific type of attribution, and the user’s causal attribution transitions can be tracked along with their behavior. Knowing the transitions in causal attribution allows us to focus on situations which deserve attention, especially by extracting shifts from them and analyzing why the shift occurred and what exactly was happening before/after the shift, namely, the situation of staying with the same attribution for a while (referred to as attribution stay). In other words, the method we propose is that the attribution shifts are used to mark the salient points, and the reasons for the marked shift and the circumstances before/after the marking (i.e., attribution stay) are checked to elicit insights for device improvement.

### Shift and Stay of Causal Attribution as Information for Improving Assistive Devices

In this study, we apply attribution theory to assess transitions (including shifts and stays) of causal attributions that occur between the 1st and 2nd test sessions of an assistive device (sessions separated by a short time interval); detailed procedures of our case study are shown in Section 4.

The occurrence of attribution shifts suggests that the user’s emotions and achievement motivation were changed or affected, and it is highly likely that this was a salient experience for the user. While it is very difficult to analyze all of the multiple user test videos over a long period of time, being able to extract the salient points would improve the efficiency of user test analysis and help in device improvement. Attribution stay, in contrast, indicates that there may be no particularly salient experience or, conversely, a fixation on a positive or negative feeling for a while. As for the latter, for instance, if one is stayed with the task difficulty, a specific operation of the device may be too difficult for the user. Thus, causal attribution stay also deserves attention since it ultimately guides the improvement of the device. For example, stay with low effort attribution may be useful to design motivational triggers for users, stay with low ability attribution suggests to design special training for handling the device, stay with high task difficulty attribution suggests to simplify the device, and stay with bad luck attribution suggests to design safety and for the elimination of external factors making the user feel bad luck.

In this way, attributions can be linked to the situation at that specific moment by classifying the actual utterances during device use. Therefore, this may allow us to identify, for example, what specific problem is causing task difficulty attribution. In other words, knowing the causal attributions during device use allows us to draw up a device improvement strategy.

## Objectives

It is especially difficult for the elderly to engage in repeated user tests. It is necessary to have a simple user testing method that can draw conclusions from user tests in the few opportunities available, even with no mastering of the user test method. In this study, we propose a method that can easily grasp transitions in users’ causal attribution related to a given test from utterance data collected during user tests of trial products and prototypes. To this end, we classify utterances collected from user tests into the four quadrants of the causal attribution framework. By knowing the transitions in causal attribution, especially by extracting the shifts from them, we aim to elucidate situations in which salient experiences that are useful for device improvement are embedded.

**Fig. 2:**
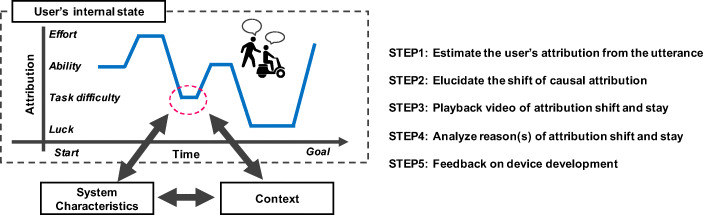
Schematic image of the proposed method and its application for device development

Figure 2 shows a conceptual diagram of the method proposed by this research and its application to device development. A user’s causal attribution is estimated from the utterance (STEP1), and the shift of the attribution is extracted (STEP2). Following this, the device designer could return to the place where the shift occurred using the recorded video and so on (STEP3), analyze why the shift occurred and the circumstances before and after it (i.e., attribution stay) (STEP4), and give feedback on the system characteristics of device development and the context of device usage (STEP5). The development of methodology in STEP1 and STEP2 is considered within the scope of this study.

## Research Methodology

To evaluate the proposed method, a user test for the Life Walker was conducted in Kakegawa City (Shizuoka Prefecture), as shown in Fig. 1 (Sub-section 4.1). In addition, all utterance data collected from drivers (frail elderly) during user tests were classified according to attribution theory (Sub-section 4.2). Considerations on how to predict transitions in emotions and achievement motivation based on the classification of utterance data according to attribution theory also considered interviews carried out after the use of the Life Walker: an electric-assisted four-wheeled cycle (Section 5). For specifications of the Life Walker device used in this study, please refer to the paper by Saijo et al. (2014).

### A Case Study in Kakegawa City, Shizuoka, Japan

**Description:**

A frail elderly person is assisted by a healthy elderly person and rides a Life Walker on a predetermined course for about 15 min.

**Date:**

November 19th, 2014.

**Place:**

Twenty-Second Century Hill Park in Kakegawa City, Shizuoka Prefecture (Tamari 1652, Kakegawa City, Shizuoka Prefecture).

**Users in the survey:**

6 pairs, each made up of a frail elderly person (driver) and a healthy elderly person (caregiver).

(Pair codes: A-F)

* To eliminate the influence of personal connections as much as possible, people who had not previously met each other were chosen.

**Data collection:**

Recording conversations during the Life Walker ride.

Recording of retrospective interviews after the test rides.

**Timeline:**

Health check (15 min).

Guidance (15 min).

1st Life Walker test ride (10–15 min).

1st retrospective interview on the ride (5 min).

2nd Life Walker test ride (10–15 min).

2nd retrospective interview on the ride (5 min).

Questionnaire survey (5 min).

**Retrospective interviews:**

After the first and second test rides, a retrospective interview was conducted for each pair of frail and healthy elderly people. Out of ten possible items, Tables 2 and 3 show the items included in the interviews that are subject to analysis in this paper.

**Table 2:** Excerpt of common items from the 1st and 2nd retrospective interviews

No.	Item	Contents
Q1	General impression	What is your impression after having “walked” with it? Please give a grade from 1 to 5 and your reasoning. (5: very good, 4: good, 3: neither good nor bad, 2: bad, 1: very bad)

**Table 3:** Excerpt of items from the 2nd retrospective interview

No.	Item	Contents
Q8	Needs related to the vehicle	Is there a place where you’d like to go with this vehicle?
Q9	Changes in impression	Was there any change in your impression after having participated in this event? Please give a grade from 1 to 5 and a reason. (5: yes, 4: a little bit, 3: difficult to say, 2: there was almost no change, 1: no)
Q10	Participation in the event again	Would you like to participate in an event like this again? Please give a grade from 1 to 5 and the reason. (5: yes, 4: a little bit, 3: difficult to say, 2: maybe not, 1: no)

### Coding Rules of Utterance Data Based on Attribution Theory

**Fig. 3:**
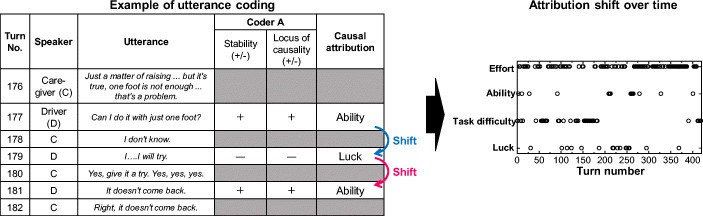
Example of utterance coding

Using Weiner’s attribution theory (two-dimensional model), all speech uttered by the frail elderly (drivers) during the test ride were classified. Utterance data was divided into turns (utterance order), which were classified into three levels along two dimensions: stability (stable/neutral/unstable) and locus of causality (internal/neutral/external). The coding rules are shown below, and an example of coding is shown in Fig. 3:

- Coding is performed according to the coding criteria of Table 4.
- Only utterances related to the driving operation are coded.
- Dialogues are considered to have an I-R-F (initiation–response–follow-up) structure, which characterizes a basic unit of linguistic interaction (Sinclair and Coulthard 1992). The coding must take into consideration the structure of the dialogue, focusing on previous and posterior utterances, and not only on the target utterance.
- If the driver’s utterance (the target of the coding) suffers an interruption because of something like back-channeling by the caregiver, giving rise to a turn change, the grammatical structure is reconstructed. If a semantic unit exists, that is considered in the coding.
- Coding is performed twice per dimension. In case of variations in the coding (for instance, “stable” the first time and “unstable” the second time for the stability dimension), the final code is defined while taking into consideration coding rules and criteria.
- If an utterance is coded as “neutral,” the corresponding dimension is considered to remain unchanged. After defining the final code, the preceding code is inherited.

**Table 4:** Coding criteria for utterance data based on attribution theory

Dimension	Code	Coding criteria	Example
Stability	Stable (+)	Utterance that gives the impression that the situation is difficult to change Utterance that demonstrates self-confidence in one’s abilities	- How clumsy! - I can’t get it yet. - I’m getting used to it.
	Unstable (−)	Utterance that gives the impression that the situation can be changed easily/has happened suddenly Utterance that demonstrates fear or surprise Asking how to do it when starting an action Utterance that recognizes that the results may change easily depending on one’s attention	- Oh, it’s OK just to go there and then come here, right? - Oops, careful. - I … I will try.
	Neutral (0)	Others	- Oh, yes.
Locus of causality	Internal (+)	Utterance that recognizes that the situation is, in any case, under his or her own control	- I will first give it a try. - Oh, it’s light.
	External (−)	Utterance that shows that the situation is, in any case, not under his or her own control Utterance that recognizes that the situation is due to an external restriction or happening	- When I trained with this (…) (my left foot) was not an artificial bone yet. - It’s heavy. - There’s a traffic jam.
	Neutral (0)	Others	- yes, thank you.

Here we see a detailed example of coding. If the driver steps on a pebble on a road and says “Look out!,” this will be attributed to the locus of causality being “external” and the stability being “unstable,” which in turn depends on “luck.” If another says “It’s difficult, I don’t know how to use it” about the usability of a small button on a device, this can be attributed to the locus of causality being “external” and the stability being “stable,” which implies “task difficulty.” In particular, the latter can be used to directly improve the device.

### Analysis Method of Collected Data

#### Considerations in the Reliability of Coding Rules

To consider the reliability of the coding rules above, two coders (the first and third authors) performed coding for 10% of all drivers’ utterances. Lombard et al. (2002) stated that at least 10% of the entirety of the data must be coded in order to investigate the reliability across different evaluators. The kappa (*κ*) coefficient was used for studying the agreement ratio. *κ* is a parameter related to agreement ratio that does not depend on chance and is expressed as follows:

$$ \kappa =\frac{N_C-{N}_e}{N-{N}_e} $$

Here, *N*_C_ is the number of coincidences, *N*_e_ is the expectation value for the number of coincidences, and *N* is the total number of data. According to Landis and Koch (1977), an agreement ratio of 0.61 or more (i.e., a substantial agreement) is the reference point in present research.

#### Considerations on the Validity of the Proposed Method

To consider the validity of the proposed method, we conducted the following three steps: (a) a qualitative discussion on emotions and achievement motivation based on retrospective interviews, (b) a qualitative discussion on the evolution of emotions and achievement motivation (especially attributions) based on excerpts of actual utterance data, and (c) a comparison between discussions (a) and (b), as well as coding results of attributions based on our proposed method to check whether the trends of attribution transitions match.

#### Considerations in Marked Points Extraction

The marked points were considered on the basis of the ratio of the number of utterances with attribution shifts to the total number of utterance data (1626 utterances). Here, the attribution shift and stay are defined as a transition from one attribution to another and staying with the same attribution over a period of time, respectively. If only users’ salient experiences can be referenced from the data, by focusing on the attribution shift and before/after it (i.e., attribution stay), useful information that can improve system characteristics and environment can be extracted in a short period of time.

## Results and Discussions

### Reliability of Coding Rules

We considered the issue of reliability across different coders by following the coding rules of Sub-section 4.2. Table 5 summarizes the number of utterances per pair and test ride. In the present research, 4128 utterances were obtained, out of which we focus on 1626 utterances that correspond to the drivers’ utterances (frail elderly). To consider reliability across different coders, we used 163 utterances by the driver of pair A (1st). This corresponds to at least 10% of the total number of 1626 utterances by the drivers, which is the target of this research.

**Table 5:** Summary of numbers of utterances in the user test

Pair number	A	B	C	D	E	F	Total
1st ride	163	126	176	160	276	74	975
2nd ride	103	78	96	121	201	52	651
Total	266	204	272	281	477	126	1626

We investigated the reliability coefficient across coding results of the first (Coder A) and third (Coder B) authors, according to Sub-section 4.2, and obtained *κ* = 0.723 for stability and *κ* = 0.706 for the locus of causality. Thus, it seems that we have successfully designed coding rules that exhibit high reliability. Following this, all driver utterances coded by the first author (Coder A) are used as coded results.

### Validity of the Proposed Method

**Fig. 4:**
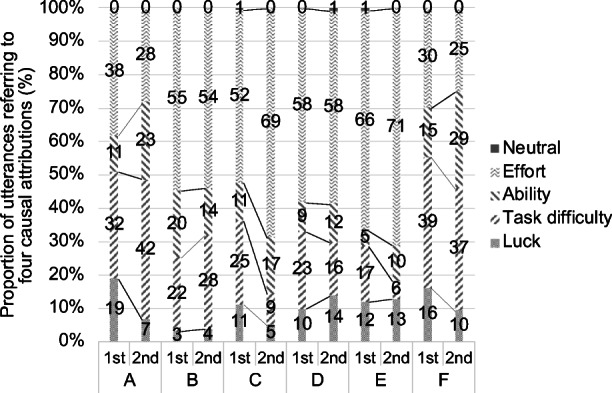
Proportion of each attribution with respect to the total number of utterances by the driver (frail elderly) during each test ride; numbers in the bars indicate respective ratios. Note that the sum may not reach 100% because of rounding to the first decimal digit

The first author performed coding on the entire set of 1626 utterances spoken by the drivers (frail elderly) based on the coding rules described in Sub-section 4.2. Figure 4 and Table 6 show the distribution of attributions of all utterances in each test ride. Note that “neutral” refers to cases coded as “neutral” in the “stability” and/or “locus of causality” dimensions, but the attribution does not fit into any of the defined causes (effort, ability, task difficulty, luck).

**Table 6:** Proportion of each attribution with respect to the total number of utterances by drivers (frail elderly) in each test ride

Pair number	A		B		C		D		E		F	
Test ride number	1st	2nd	1st	2nd	1st	2nd	1st	2nd	1st	2nd	1st	2nd
Effort	38%^a^	28%	55%	54%	52%	69%	58%	58%	66%	71%	30%	25%
	62^b^	29	69	42	91	66	93	70	182	143	22	13
Ability	11%	23%	20%	14%	11%	17%	9%	12%	5%	10%	15%	29%
	18	24	25	11	20	16	14	14	13	21	11	15
Task difficulty	32%	42%	22%	28%	25%	9%	23%	16%	17%	6%	39%	37%
	52	43	28	22	44	9	37	19	46	11	29	19
Luck	19%	7%	3%	4%	11%	5%	10%	14%	12%	13%	16%	10%
	31	7	4	3	20	5	16	17	33	26	12	5
Neutral	0%	0%	0%	0%	1%	0%	0%	1%	1%	0%	0%	0%
	0	0	0	0	1	0	0	1	2	0	0	0
Number of utterances by the driver	163	103	126	78	176	96	160	121	276	201	74	52
Total number of utterances	409	241	321	231	495	285	420	303	658	437	187	141

^a^Percentages indicate respective ratios; note that the sum may not reach 100% because of rounding to the first decimal digit

^b^The numbers in the table indicate the frequencies of each attribution from the total number of utterances by the driver

According to Fig. 4 and Table 6, if we consider changes from the first to the second test ride, we can see that the ratio of “effort” attribution in the second test ride went down for pairs A, B, and F, and the ratio of the “difficulty of the task” attribution went up (except for a slight drop of 2% for F). In contrast, the ratio of “effort” attribution increased for pairs C, D, and E (with no change to D), while the ratio of the “difficulty of the task” decreased. For the three pairs in the former case, it is possible that the causes for the results of the riding experience were attributed to an external factor, especially the Life Walker itself. With regard to the three pairs from the latter case, the causes may be attributed to an internal factor (the driver themselves), which would have resulted in repeated trial-and-error driving actions.

Below, we consider the examples of pairs A and D and investigate the relations between the proposed method and observed emotions and achievement motivation (the remaining pairs are described in the Appendix Figures 9, 10, 11, 12).

#### Considerations on pair A:

The emotions and the achievement motivation of the driver in pair A is considered in the form of a retrospective interview. Some results of the retrospective interviews in Tables 2 and 3 are summarized below (Table 7).

**Table 7:** Excerpt of the results of the 1st and 2nd retrospective interviews for pair A

No.	Item	Answer
Q1 (1st)	General impression	5-grade evaluation: 2 (bad) *Heavy. Tiring.*
Q1 (2nd)	General impression	5-grade evaluation: 3 (neither good nor bad) *Not so good that it deserves a compliment.*
Q8	Needs related to the vehicle	*I wouldn’t like to ride it at all.*
Q9	Changes in impression	5-grade evaluation: 1 (no) Not Available (N/A)
Q10	Participation in the event again	5-grade evaluation: 3 (difficult to say) N/A

The driver in pair A said things like “heavy” and “I wouldn’t like to ride it at all,” which suggests negative emotions and low achievement motivation with respect to the device usage. We consider how attributions changed for the driver in pair A from the viewpoint of attribution theory. Below are quotes extracted from the actual utterances; Numbers refer to turn numbers, parentheses indicate speakers, and the text describes the actual utterances.

Excerpt A-1-1

*56. [Caregiver] Is it heavy?*

*57. [Driver] No, it’s not. No, I don’t think it’s heavy.*

*171. [Driver] Oh, it’s light.*

*177. [Driver] It feels good.*

Unlike the results of the retrospective interview, the impression of being “light” was present in the first half of the ride, and therefore it can be said that the operation of the Life Walker resulted in a high level of self-efficacy.

Excerpt A-1-2

*200. [Others] It’s true. The button operation is good too.*

*203. [Driver] I can’t get it yet.*

We can see signs that complex operations involving buttons and other things are starting to be perceived as overwhelming.

Excerpt A-1-3

*231. [Caregiver] Your handle operation is good.*

*242. [Driver] It’s too heavy.*

*250. [Driver] It’s still heavy.*

*300. [Driver] It’s heavy; it’s heavy. You see, this is terrible.*

*370. [Driver] It’s heavy; it’s heavy. It’s heavy.*

We can see that the driver is looking to the external element, the Life Walker, as the locus of causality, even after the caregiver gave encouragement. Moreover, it seems that being “heavy” is a fact that is difficult to change, which leads to the attribution “task difficulty.”

From the above, it seems that the attribution shifted with time from “effort” to “ability” and then to “task difficulty” (as operation of the Life Walker was repeated), resulting in lower achievement motivation. Negative expressions regarding the device such as “heavy” and “I wouldn’t like to ride at all” during the retrospective interviews may be a result of this shift.

Next, we discuss the relationships between the achievement motivation of the driver of pair A, which is qualitatively estimated from the retrospective interviews and utterance data, and the results of the proposed method. In Table 6, we observe a reduction from 38% to 28% in the ratio corresponding to the “effort” attribution from the 1st to the 2nd test, and an increase from 32% to 42% in the ratio corresponding to the “task difficulty” attribution. It has been pointed out that in situations of failure, the “effort” attribution contributes to enhanced motivation (Dweck 1975). In the case of these user tests, the driver is supposedly subject to multiple situations of failure, and therefore it is reasonable to think that shifts in achievement motivation can be estimated by observing ratio variations and the evolution of the “effort” attribution. In the case of the driver in pair A, it seems that an external element was regarded as the causing factor, and this fact contributed to the depreciation of achievement motivation.

**Fig. 5:**
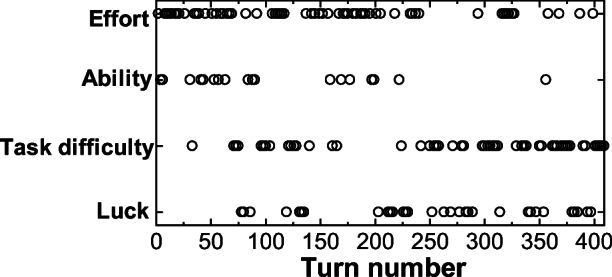
Attribution transitions in the utterances of the driver in pair A (1st)

**Fig. 6:**
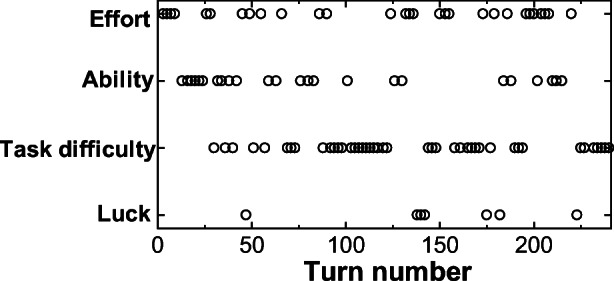
Attribution transitions in the utterances of the driver in pair A (2nd)

We will investigate further how the attribution of the driver in pair A changed over time. Figures 5 and 6 show the evolution of attributions in test rides 1 and 2. We can clearly see, especially in Fig. 5 (1st test ride), that the “effort” attribution that existed in the first half was replaced by an “ability” attribution, and then finally shifted to the “task difficulty” attribution. This trend of transitions in attributions (Figs. 5 and 6) matches the above-mentioned qualitative discussion and the several excerpts of actual utterances.

#### Considerations on pair D:

The emotions and the achievement motivation of the driver in pair D are considered in the form of a retrospective interview. Some results of the retrospective interviews are summarized in Table 8.

**Table 8:** Excerpts of the 1st and 2nd retrospective interviews for pair D

No.	Item	Answer
Q1 (1st)	General impression	5-grade evaluation: 2 (bad) *It hurts when I turn the pedal.*
Q1 (2nd)	General impression	5-grade evaluation: 4 (good) *Reversing is a problem. It’s difficult to use the pedal.*
Q8	Needs related to the vehicle	*None*
Q9	Changes in impression	5-grade evaluation: 5 (yes) *It’s fun to run watching the outside landscape. At home, I can hardly walk 300 m using a cane. It’s good to pedal with my own feet.*
Q10	Participation in the event again	5-grade evaluation: 5 (yes) N/A

The driver in pair D has an artificial bone in one knee. In the retrospective interview, she mentions the difficulties involved with pedaling. However, when asked about general impressions after the second test ride, Q1 changed to a positive value. She then says that it was fun to be able to “pedal with my own feet” and “watch the outside landscape.” From the above, it seems that her emotions changed for the positive, and the achievement motivation with respect to the device usage increased during the event.

Next, we consider how attributions changed for the driver in pair D from the perspective of attribution theory. Below are representative scenes extracted from actual utterances.

Excerpt D-1-1

*153. [Driver] I can see that it’s too much for me.*

*155. [Driver] When I trained with this, well, my left foot still ... well ...*

*157. [Driver] It was not an artificial bone yet.*

*167. [Driver] Well, yes. If I could, I would just turn it and run like this ... vrrr ...*

This suggests that external restrictions (the artificial bone is a fact that cannot be changed) make it impossible to get comfortable with the vehicle, and the reason is input as part of “task difficulty.”

Excerpt D-1-2

*168. [Caregiver] Yes, you’d like to ride, right?*

*173. [Driver] Yes. Yes. But then, you see, look at my leg, how I raise my knee.*

*176. [Caregiver] Just a matter of raising ... but it’s true, one foot is not enough ... that’s a problem.*

*177. [Driver] Can I do it with just one foot?*

*178. [Caregiver] I don’t know.*

*179. [Driver] I….I will try.*

*180. [Caregiver] Yes, give it a try. Yes, yes, yes.*

*181. [Driver] It doesn’t come back.*

*182. [Caregiver] Right, it doesn’t come back.*

The caregiver often accepted the driver’s observations. Based on these acceptances, the driver tries to perform operations with only one foot but fails. From this, we can verify that the lack of “ability” is being recognized as a barrier.

Excerpt D-1-3

*278. [Caregiver] You’re getting used to it.*

*279. [Driver] Yes, I am.*

Starting from the driver’s turn number 278, the number of utterances evaluating the driver’s own driving (self-monitoring) suddenly increases (refer to the following excerpt).

Excerpt D-1-4

*357. [Driver] Going there …*

*359. [Driver] Moving away a little bit ...*

*361. [Driver] Changing direction …*

*363. [Driver] Yes, moving away …*

*365. [Driver] Moving away a little bit and changing direction.*

Spontaneously performing self-monitoring is an indication that the driver is starting to recognize that she is responsible for the results (locus of causality: internal) and that the success or failure of driving may change depending on her attention (stability: unstable). Therefore, we can classify the attribution of this excerpt as “effort.”

From the above, we can conjecture that the driver in pair D is strongly influenced by verbal persuasion, such as being indulged when something is unachievable or getting a compliment, which resulted in an attribution shift from “task difficulty” to “ability” and then to “effort.” In the 2nd retrospective interview, the driver said things such as “because the caregiver encouraged me, saying that I’m good” or “you feel more comfortable now”; this confirmed the assumption that there exists a strong influence of verbal persuasion in the caregiver’s argumentation. We can also observe several instances of spontaneous self-monitoring in the second test ride, that kept the achievement motivation at a high level with respect to device usage. Moreover, we can see comments such as “the air outside was pleasant” during the retrospective interview, which may be an indication that the situation led to a positive perception of the event as a whole.

In the following, we discuss the relation between the achievement motivation of the driver in pair D, estimated from a qualitative discussion based on retrospective interviews and utterance data, and the results of the proposed method.

Comparing the ratios that correspond to the “effort” attribution in Table 6, we can see that it remained at 58% for both the 1st and 2nd tests. The “ability” attribution changed from 9% to 12%, the “task difficulty” attribution changed from 23% to 16%, and the “luck” attribution changed from 10% to 14%. As a result, the ratio of “internal” as the locus of causality increased in the second test, which suggests an internalization of the operations related to the device. However, it also suggests that a change in the ratio of the “effort” attribution, such as the one observed for the driver in pair A, does not necessarily lead to a conjecture of shifts in achievement motivation.

**Fig. 7:**
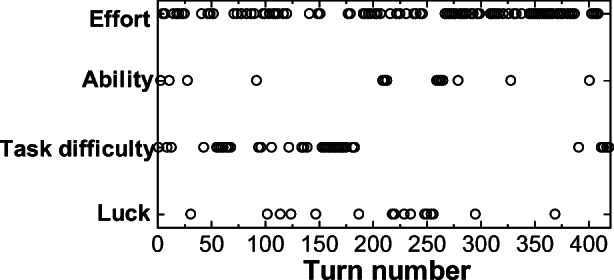
Attribution transitions for the utterances of the driver in pair D (1st)

**Fig. 8:**
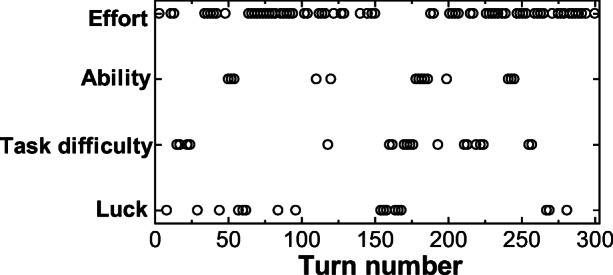
Attribution transitions for the utterances of the driver in pair D (2nd)

The coding results for the first and second test rides are shown in Figs. 7 and 8. We can see in Fig. 7 (1st test ride) that the “task difficulty” and “effort” attributions alternate repeatedly in the first half. In the second half, we can see that the “effort” attribution appears more frequently, becoming the main attribution in Fig. 8 (second test). The trend of transitions in attributions (Figs. 7 and 8) matches the above-mentioned qualitative discussion and the several excerpts of actual utterances.

#### Summary of this sub-section:

In this sub-section, we performed comparisons involving retrospective interviews, actual utterance data, and the coding results of the proposed method. A comparison between retrospective interviews and actual utterance data suggests that the emotions and achievement motivation with respect to device usage, which are manifested in retrospective interviews, may depend on the second half of each test ride. Moreover, a comparison between qualitative discussions on actual utterance data and attributions extracted from the coding shows an approximate match, which suggests the validity of the results obtained from utterance coding based on the coding rules of Sub-section 4.2.

### Marked Points Extraction from Utterance Data

Table 9 shows the ratios of attribution shifts with respect to all utterances of the user tests collected in the present research. In the present research, attribution shifts were observed in 540 of the total number of utterances (1626), which is about 33%.

**Table 9:** Frequencies of attribution shifts and ratios with respect to the total number of utterances

Pair number	Test ride number	Total number of utterances	Attribution shift
A	1st	163	72 (44%)
	2nd	103	47 (46%)
B	1st	126	29 (23%)
	2nd	78	25 (32%)
C	1st	176	49 (28%)
	2nd	96	22 (23%)
D	1st	160	56 (35%)
	2nd	121	40 (33%)
E	1st	276	93 (34%)
	2nd	201	62 (31%)
F	1st	74	34 (46%)
	2nd	52	11 (21%)
Total		1626	540 (33%)

The utterance data handled in this research is comprised of 1626 utterances, which is a considerable amount of data. Moreover, unlike utterances from interviews or discussions, they are made up of fragmented phrases attached to the execution of a given task. Yet, using the proposed method, it was possible to reduce the points to be considered to about 33% of the total. In addition, the utterances corresponding to these 33% correspond to shifts in attributions related to the task due to the influence of system characteristics of the device as well as usage context. It is highly possible that, for the driver, this represented a salient experience. Now, it is necessary to deepen our investigation into the scenes where attribution shifts occurred and before/after them (i.e., attribution stay). In the present method, flags have been planted to signal particular experiences for future reference. Thus, we expect that this method will extract useful information to help improve system characteristics and use contexts.

## Conclusions and Future Direction

### Conclusion of this Study

In this study, we aimed to establish a methodology that gives useful insight and feedback to assistive device development from limited user testing opportunities in the context of the elderly. By focusing on the attribution that is unconsciously performed by users, we proposed a method to classify all utterances of device users into two dimensions (“stability” and “locus of causality”) and four perceived determinants of achievement behaviors (“effort,” “ability,” “task difficulty,” and “luck”). In doing so, we attempted to extract transitions in attributions, especially attribution shifts to mark the salient experiences of users during device usage. Following this, the reasons for the marked shift and circumstances before/after the marking (i.e., attribution stay) were checked to elicit insights for device improvement. Although it is always challenging to analyze multiple user tests’ data over long periods of time, our proposed method is able to extract the salient points from user tests, which would allow us to improve the efficiency of user test analysis and effectively improve the device.

To validate our proposed method, we conducted the user test of an electric-assisted four-wheeled cycle (Life Walker) for frail elderly people in Kakegawa city, Shizuoka, Japan. Considerations were made on the relation between the coding results of utterance data based on the proposed method, and emotions and achievement motivation extracted through other methods involving retrospective interviews and actual utterance data. By comparing retrospective interviews and actual utterance data, it was found that the achievement motivation that is manifested in retrospective interviews with respect to the device may depend mostly on the second half of the test ride. Moreover, a comparison between qualitative discussions on attribution transitions based on actual utterance data and attribution transitions extracted from the coding shows an approximate match, which suggests the validity of the results obtained from utterance coding based on the coding rules of the proposed method. In addition, in using the proposed method, we succeeded in marking the points that require attention, which are about 33% of the total amount of utterance data (1626 utterances), thus confirming the potential of our proposed method.

In particular, these results suggest that the proposed method can be used to improve devices more efficiently by extracting only the situations that include salient experiences, rather than analyzing the meaning of all utterances.

### Limitations and Future Study

In this research, we discussed several excerpts to assess the validity of the proposed method, although we did not analyze every single situation in which attribution shift/stay occurred. It may be necessary to consider the relations between the situations extracted by the proposed method and their respective attributions. We also need to account for any combinations/patterns of attribution transitions, which might be affected by various factors including the user’s learning strategies and the characteristics of the device itself. Moreover, a possible extension of the method could be to apply the coding to not only the driver, but also other people’s utterances (e.g., the caregiver’s). In the test ride, we observed the strong influence of verbal persuasion by a caregiver on the driver’s achievement motivation (see the 1st test ride of pair D). This kind of phenomenon can be systematically analyzed by expanding our proposed method to other people’s utterances.

Also, as described in Section 3, there are five steps to utilize the proposed method to improve devices, two of which were examined in this study. Future research should examine the remaining three steps to see if using this methodology could help designers improve their products.

In addition, the coding of utterance data in the present research was performed manually by the first author, and this task consumed a considerable amount of time. From now on, if the number of cases to use as training data increases to a level that permits machine learning, the range of applications will expand dramatically. The present method was designed to account for classification in the two dimensions of stability and locus of causality, not the four categories of effort, ability, task difficulty, and luck. This could allow us to use machine learning in future research. In natural language processing, for example, it is common to process classifications into two classes such as positive/negative. Similarly, such an approach could constitute a possible line of future research.

Finally, there are many situations, like the user test in the present research, where utterances naturally occur in the process of achieving the task. If people can identify the attribution of an event that is taking place, they may be able to intervene and change that attribution, which can result in increased motivation. The possible applications of this could extend to several situations in fields including education and social assistance.

## Figures and Tables

**Fig. 9:**
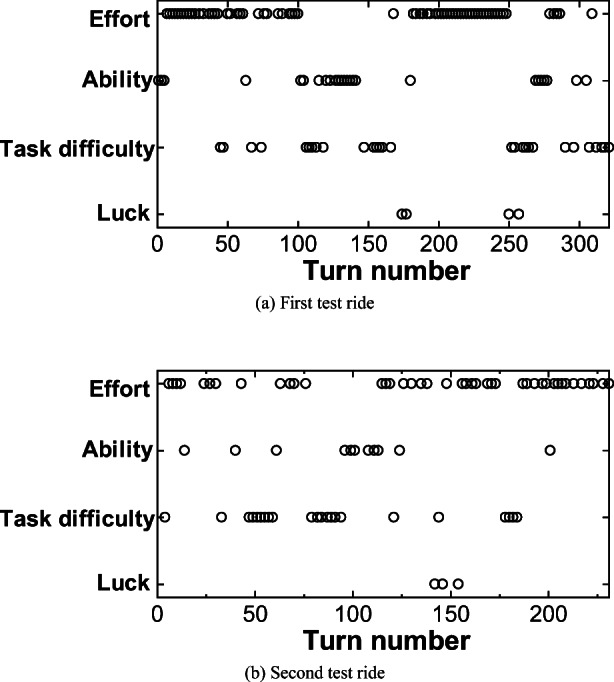
Attribution transitions in the utterances of the driver in pair B

**Fig. 10:**
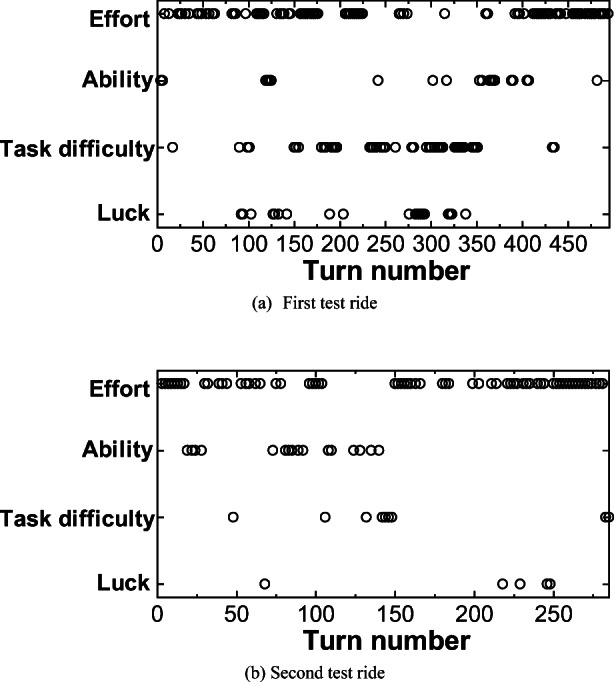
Attribution transitions in the utterances of the driver in pair C

**Fig. 11:**
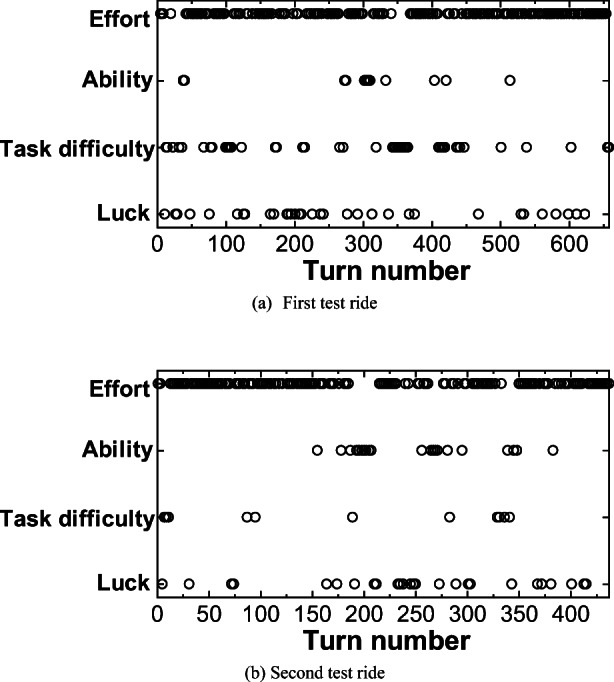
Attribution transitions in the utterances of the driver in pair E

**Fig. 12:**
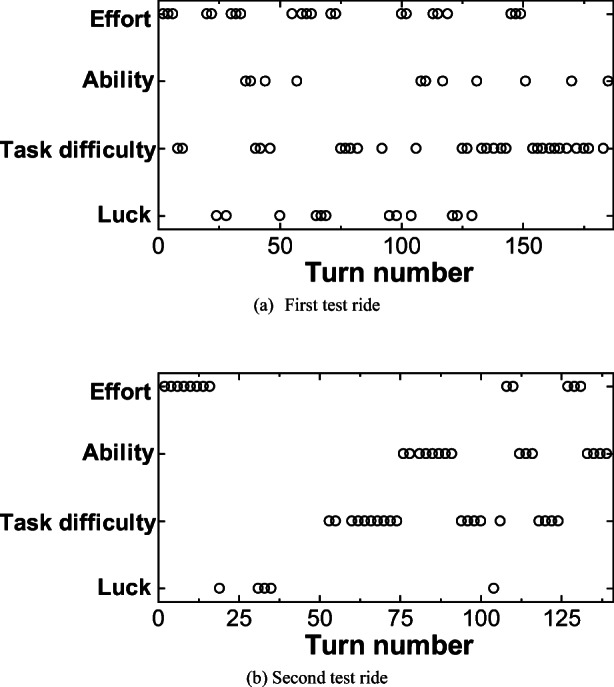
Attribution transitions in the utterances of the driver in pair F

## Appendix

The figures below show the changes in attributions related to the driver’s utterances that were not discussed in the paper.
